# Polyhydroxyalkanoates, bacterially synthesized polymers, as a source of chemical compounds for the synthesis of advanced materials and bioactive molecules

**DOI:** 10.1007/s00253-021-11589-0

**Published:** 2021-09-18

**Authors:** Maciej W. Guzik

**Affiliations:** Jerzy Haber Institute of Catalysis and Surface Chemistry Polish Academy of Sciences, Niezapominajek 8, 30-239, Kraków, Poland

**Keywords:** Polyhydroxyalkanoates, Biomaterials, Regenerative medicine, Sugar esters, Deep eutectic solvents

## Abstract

Research into polyhydroxyalkanoates (PHAs) is growing exponentially. These bacterially derived polyesters offer a spectrum of possible applications, such as in manufacturing of daily-use objects, production of medical devices and implantable objects, or as synthons in chemical and pharmaceutical industries. Thanks to their broad physicochemical features, PHAs can be seen as polymers of the future, which can replace traditional petrochemical equivalents. As they are synthesized by bacteria through fermentation processes, these polyesters can be obtained from virtually any carbon source in a sustainable manner. Characterized by biodegradability and biocompatibility, they are used in many industries, ranging from production of everyday objects to medical applications. Furthermore, as they are built from bioactive monomers, namely (*R*)-3-hydroxyacids, they provide a platform for the synthesis of advanced chemical compounds. In this mini review, the reader will be acquainted with recent studies conducted at the Jerzy Haber Institute of Catalysis and Surface Chemistry of the Polish Academy of Sciences in collaboration with other groups that have contributed to the development of PHA-based medical materials, bioactive molecules and novel green solvents derived from PHA monomers.

**Key points**

• *Polyhydroxyalkanoates are emerging polymers for biomedical applications*

• *Polyhydroxyalkanoates can be modified easily to provide novel materials*

• *(R)-3-Hydroxyacids are good synthons for bioactive substances and green solvents*

## Introduction

Nature offers a range of polymers with numerous biological, chemical, and physical properties, including plant polymers—starch, cellulose or lignin, and animal polymers such as collagen, chitin or chitosan. Biopolymers are also common in bacteria and archaea. Some interesting polymers synthesized by these organisms are polyhydroxyalkanoates (PHA). They were first described in 1926 by Lemoigne, who showed that the unknown material accumulated by *Bacillus megaterium* is a homopolyester of (*R*)-3-hydroxybutyric acid [P(3HB)] (Lemoigne 1926). Polyhydroxyalkanoates are biodegradable, optically active polyesters. They can be accumulated by bacteria under unbalanced growth conditions, namely, when nutrients such as nitrogen, phosphorus, sulphur, oxygen or magnesium compounds are restricted (Muhammadi et al. 2015; Raza et al. 2018a, b). These materials accumulate as intracellular hydrophobic granules (carbonosomes), which are usually 0.2–0.7 µm in diameter and consist of 97.7% PHA, 1.8% proteins and 0.5% lipids (Koller et al. 2010). Thanks to the use of different carbon substrates, culture conditions and careful selection of bacterial species, many PHA monomers have been identified. More than 150 hydroxyacids are present as components of PHA (Steinbüchel and Valentin 1995; Dinjaski and Prieto 2015), which makes them the largest group of natural polyesters (Steinbüchel and Valentin 1995). Moreover, more monomers are constantly being discovered through modifications of their structure by chemical synthesis.

**Fig. 1:**
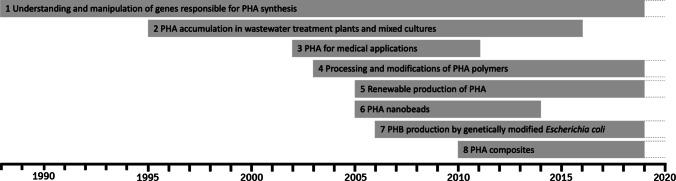
Eight identified trends in polyhydroxyalkanoate research [reproduced from Guzik et al. (2020)

The diversity of the PHA polymer family has resulted in an exponentially increasing interest in these compounds in the scientific and industrial circles since the 1970s. Most PHA representatives have two extremely attractive features, namely, their biocompatibility and biodegradability, which offer broad possibilities for applications, both in everyday life and in medicine. In a published systematic review, we described the past and present trends in polyhydroxyalkanoate research (Guzik et al. 2020). Using advanced algorithms, such as HDBSCAN (*Hierarchical Density-Based Spatial Clustering of Applications with Noise*) together with scientists from the Cracow University of Economics, we studied a collection of 2227 full text-based original PHA research papers from the Web of Science Core Collection. This sample revealed eight trends defining key directions of research in this field to emerge (Fig. 1).

The main research trends aim to clarify the mechanisms of PHA biopolymer synthesis. New genes and their combinations in various microorganisms, used to produce biochemical apparatus to synthesize PHA (trend 1), are constantly being discovered. To understand their role in PHA accumulation, these genes are transferred to well-known organisms such as *Escherichia coli* (trend 7), resulting in new and efficient genetically modified strains tailor-made for PHA polymer synthesis. Another aspect, which exists on the borderline of genetics, biochemistry and physiology of microorganisms, concerns understanding carbonosomes. These are granules in which PHA is deposited, and they are potentially useful in biochemistry, immunology and medicine (trend 6).

Equally important are topics described in literature, such as the synthesis and applications of PHA. Herein, these are the two most important aspects. Great effort has been exerted to understand PHA synthesis in microorganisms. Two schools exist: The first one assumes the superiority of genetic manipulation of selected production strains to maximize biomass and polymer content, and one example is the research described in trend 7. Such an approach certainly can provide increased efficiency; however, applying these developed technologies in individual countries is problematic—for instance, countries in the European Union have placed limitations on the use of genetically modified organisms. Despite this, novel PHA production technologies that employ wild-type strains are under development, guided by the ideas of green chemistry and sustainable development. These include processes optimized for PHA synthesis in sewage treatment plants based on mixed bacterial cultures (trend 2) or those using monocultures with renewable carbon and energy sources (trend 5).

Herein, the reader will be acquainted with the potential applications of medium-chain-length PHA polymers. Newly developed PHA-based materials show promise for medical applications, whether in exclusively polymeric or hybrid forms. One prominent example discussed in this mini review will be the results of research showing the promise of these materials as dressings and bone implants (Cichoń et al. 2019; Witko et al. 2019, 2020; Majka et al. 2020a, b).

The potential applications of PHAs may go further. These polymers are made of optically active and chiral (*R*)-3-hydroxyacids. These molecules have two easily modifiable chemical groups—carboxylic and hydroxylic—making them potentially useful in broadly understood chemical synthesis. For example, attaching a short anticancer peptide to (*R*)-3-hydroxyacids significantly increases their biological activity (O’Connor et al. 2013; Szwej et al. 2015). Compounds based on (*R*)-3-hydroxyacids show anti-microbiological activity, as shown with a panel of pathogenic bacteria and yeasts (Radivojevic et al. 2016). Accordingly, we developed methods of biocatalytic synthesis for a new family of (*R*)-3-hydroxyacid-based compounds, namely fatty acid sugar esters (Staroń et al. 2018; Snoch et al. 2019b). This mini review will also discuss organic synthesis-enabling modification of the (*R*)-3-hydroxyl group of PHA monomers by substituting ether with fluorine-containing small molecules (Snoch et al. 2019a). The compounds synthesized thusly can be used in the cosmetic, pharmaceutical and food industries, not only because of their antimicrobial or anticancer properties, but also because of their surface-active characteristics (Snoch et al. 2019a). Another interesting aspect, with high application potential in selective extraction of biomass components, is applying PHA monomers to synthesize new green solvents. The synthesis of deep eutectic solvents (DESs), in which the hydrogen bond donors are (*R*)-3-hydroxyacids, will be presented. Selected aspects of physical properties of synthesized DESs based on PHA monomers and choline, 1-ethyl-3-methylimidazole or tributylmethylammonium chlorides will also be discussed (Haraźna et al. 2019; Archer et al. 2020).

## Application of P(3HO) in the design of materials for medicine

### Examples of P(3HO)-based materials and their synthesis paths and material characteristics

#### Synthesis of P(3HO)-based dressings

The market for dressings is one of the most rapidly developing areas of medical supplies. In 2020, it was valued at approximately USD 7.0 billion, and it is predicted to reach USD 11.2 billion in 2025, at a sustained annual growth rate of 9.7% (MarketsandMarkets 2020). There is also a strong demand for the synthesis of biocompatible products from renewable carbon sources. PHA-based substances show promise for these two trends. Therefore, we have started to synthesize such materials based on elastomeric P(3HO).

**Fig. 2:**
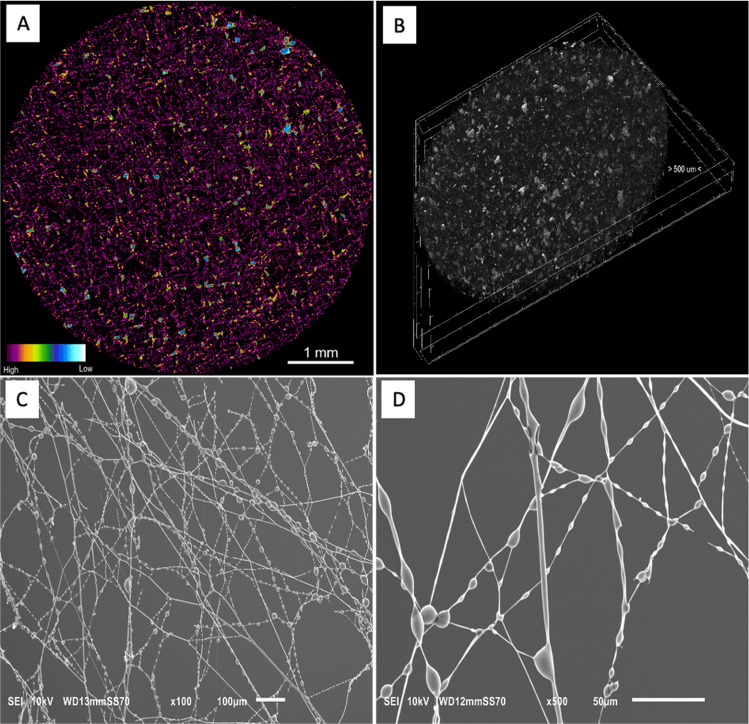
Synthesis of P(3HO)-based dressings. Reconstructed cross-section of foam obtained from 5% P(3HO) solution (**A**) and its three-dimensional representation (**B**) (pictures by Bartosz Leszczyński and Andrzej Wróbel, Jagiellonian University). SEM images of the P(3HO) fibres obtained by electrospinning (**C**, **D**, pictures by Edyta Hebda, Cracow University of Technology)

The first method developed was the solvent casting—porogen leaching methodology (Tran et al. 2011). We used sifted NaCl grains of 100–300 µm in size as a porogen. We also experimentally selected the concentration of P(3HO) solution in ethyl acetate, which enabled the polymer to effectively and evenly coat the NaCl grains. After evaporating the solvent, the salt grains were rinsed away, yielding a thin, soft foam. These materials were subjected to computed microtomography (µCT) to visualize the resulting pores and verify if there are any connecting channels between them (open porosity) and to analyse the porogen washing process. The study showed that for the 5% P(3HO) solution used, the average size of the pores was 179 µm with 98% porosity (Fig. 2A, B). However, not all pores had channels for proper rinsing of sodium chloride, and the microphotographs show its negligible residue (light-blue areas).

The second method used was electrospinning. In cooperation with scientists from the Cracow University of Technology, we attempted to spin nanofibres from the solution. The selected initial conditions did not realize a material with favourable characteristics—the fibres instantaneously agglutinated on the collector, creating a layer of P(3HO) film. Our original idea was to use a coolant, namely liquid nitrogen, in the spinning process, which allowed us to obtain fibres of nanometric diameter (Fig. 2C, D) (Majka et al. 2020a). SEM observation of the electrospun fibres revealed beads on their structure. Unfortunately, eliminating this type of fibre by manipulating the process parameters was unsuccessful. Blowing the surface of the harnessed mat with compressed air shortens the crystallization time of the polymer from about 2 weeks to 3 h (Majka et al. 2020b).

#### Synthesis of bone implants—composite ceramic-polymer materials

The development of composite bone substitute materials is another rapidly growing branch of regenerative medicine. Such composites should show biocompatibility, bioactivity, osteoconductivity, or even osteoinductivity to promote the growth of new tissues (Velasco et al. 2015). Their architecture must also encourage cell migration and proliferation and, in addition, ensure an adequate distribution of nutrients that directly affect the regeneration of damaged bone tissue. Some advantageous materials used in the regeneration of bone tissue are those based on phosphate-calcium ceramics (i.e. hydroxyapatite, α/β-tricalcium phosphates), which largely meet the previously presented requirements, especially when used in the form of porous scaffolds. Such ceramic scaffolds can be given additional functions. One strategy is to coat them with a biodegradable polymer—this not only improves their mechanical properties, but often also introduces the possibility of using them as controlled delivery systems.

Many synthetic (e.g. polycaprolactone, poly[lactic-co-glycolic acid], poly-dl-lactide) and natural (e.g. scl-PHAs, collagen, alginate, or silk) polymers were proposed as coatings for ceramic scaffolds (Wang et al. 2005; Nikkola et al. 2015; Edgar et al. 2016; Mota et al. 2017; Araújo et al. 2017; Ang et al. 2020). P(3HO) had only been studied as a material for soft tissue engineering, while the applications of this polymer in bone tissue engineering were not proposed by other groups. Therefore, this novel elastomeric polymer opens new routes for prolonged nourishment of the regenerating tissue, showing the promise of this platform for further functionalization with bioactive compounds for controlled drug release systems. To tap into this potential, together with colleagues from the AGH University of Science and Technology in Kraków, Poland, we have developed tricalcium phosphate (V) (TCP) matrix materials coated with a polymer—polyhydroxyoctanoate (P[3HO])(Cichoń et al. 2019). Ceramic scaffolding was made from highly reactive powdered TCP obtained by the wet preparation method (Ślosarczyk and Paszkiewicz 2005). The β-TCP powder was used to prepare three-dimensional macroporous sponges. Using µCT, it was possible to assess the architecture of the materials, and the efficiency of the polymer coating, and to estimate the pore size in the scaffold. Uncovered ceramic scaffolds had an average pore size of 416 ± 252 µm, while that of materials coated with P(3HO) was 254 ± 45 µm. The polymer coating allowed the material to maintain its integrity even after compression strength tests. However, the presence of P(3HO) did not significantly affect the stress–strain characteristics of the materials—the compressive strength values of uncoated and polymer-coated materials were 6.0 ± 1.8 MPa and 6.7 ± 2.5 MPa, respectively. We have also conducted tests of chemical stability, in vitro bioactivity and degradation of the obtained materials. After 21 days of incubation in a simulated body fluid (SBF), we observed the formation of an apatite layer on the surface of TCP/P(3HO) materials, with only single apatite precipitations on the surface of TCP ceramics, confirming that the P(3HO) elastomeric coating on TCP favours the formation of a pseudo-apatite layer. During incubation, the composites gradually degraded. Analysis of the SEM images revealed microcracks and cavities in the polymer layer. This was confirmed by HPLC–MS/MS analyses that proved the release of P(3HO) monomers and oligomers into the SBF solution (Czechowska et al. 2021).

### In vitro biological evaluation of manufactured materials

#### Effects of P(3HO) on cytoskeleton architecture, fibroblast migration and wound healing processes in an *in vitro* model

**Fig. 3:**
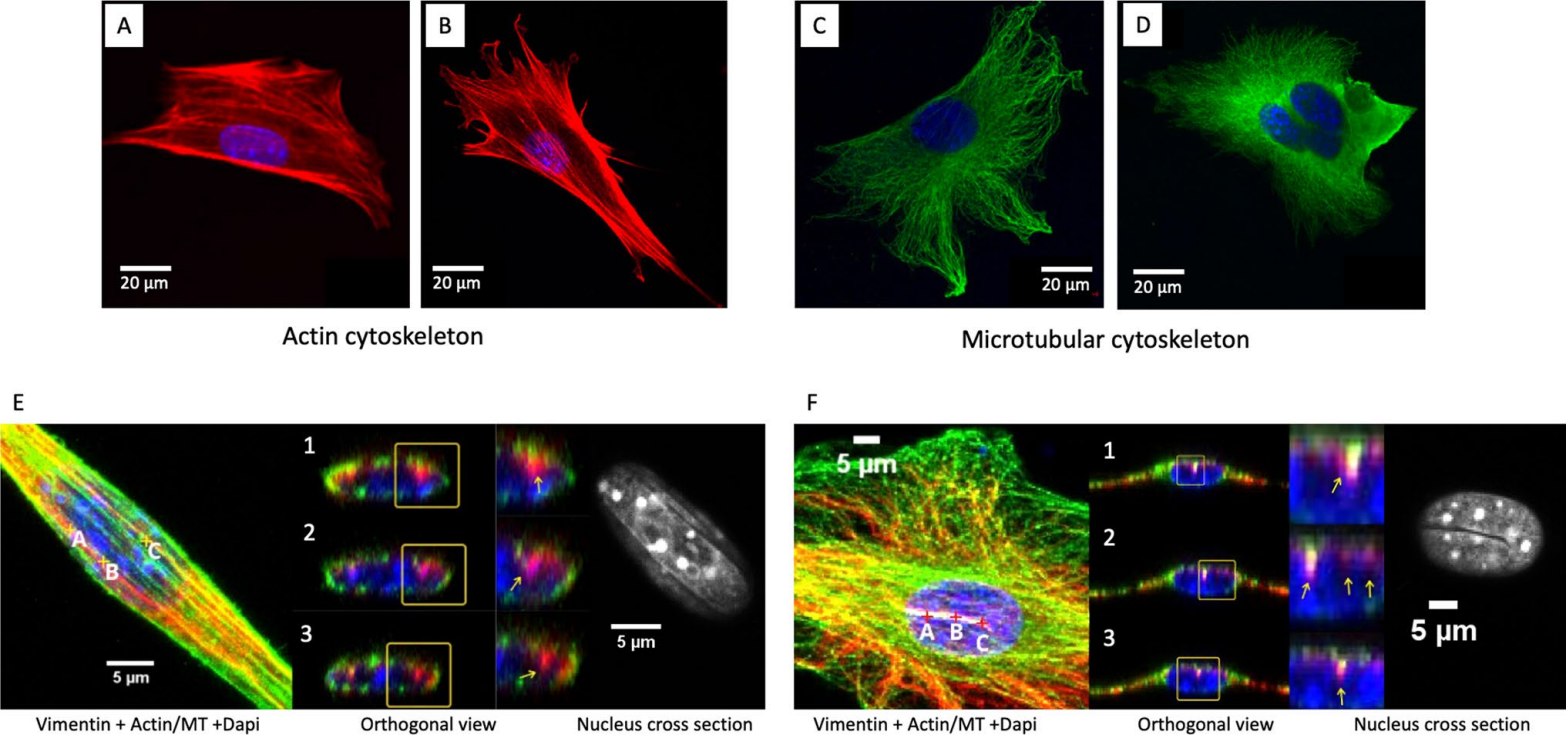
Comparison of actin (red) and microtubular (green) cytoskeleton for MEF 3T3 cells cultured on different substrates (glass—**A** and **C**; P(3HO)—**B** and **D**)—visible differences in morphology [adapted from Witko et al. (2019)]. Comparison of grooves for selected MEF 3T3 cells cultured on P(3HO) (**E**) and glass (**F**), where cells were stained for actin or microtubules (green), vimentin (red) and DAPI (blue) (adapted from Feliksiak et al. (2020))

To assess their use in wound treatment, flat materials made of P(3HO) have undergone in-depth biological characterization using the MEF 3T3 line of embryonic mouse fibroblasts (Witko et al. 2019, 2020; Feliksiak et al. 2020). To determine the cytotoxicity of this method, P(3HO) films were prepared by pouring a polymer solution from three solvents (acetone, ethyl acetate and chloroform), which were subsequently used as culturing substrates for MEF 3T3 embryonic mouse fibroblasts. The research at Jagiellonian University revealed that the solvent has no significant effect on cell proliferation, but the time elapsed since the preparation of growth substrate is important. Fibroblasts grew normally on materials as early as 24 h after their preparation, regardless of the ageing process of the material; substrate stabilization took about 2 weeks. Using advanced confocal microscopy, we visualized the cytoskeletal architecture of fibroblasts grown on P(3HO) films. The cells presented a varied morphology (Fig. 3(A–D)). Compared to the control (glass), the fibroblasts grown on P(3HO) film were more convex—their average height was 6–12 µm compared to 4–6 µm for glass. Numerous tiny branches of actin and microtubule fibres were visible, which differed significantly from those of the cells grown on glass. However, when the vimentin cytoskeleton was observed, half of the cells’ population had grooved or ‘coffee bean’–like nuclei, similar to those grown on glass (Fig. 3(E, F)). We also developed a method of quantitative comparative analysis of selected cell regions using ImageJ algorithms. The research showed that the total area occupied by actin in cells growing on P(3HO) was significantly smaller than those growing on glass. Measurements of microtubule content revealed an opposite trend. Together, these observations led to the hypothesis that P(3HO) may be a valuable material for medical appliances.

Morphological observations and quantitative results of the content of cytoskeletal components were correlated with migration parameters of fibroblasts on the tested media (Witko et al. 2019). The migration rate of MEF 3T3 cells on P(3HO)-based materials was significantly slower than that for the control group: 0.35 µm/min and 0.90 µm/min for P(3HO) poured from acetone and glass, respectively. This result directly explains the lower density of polymerized actin in these cell populations. The step size with which the cells migrated to biopolymer substrates was also significantly lower, namely, 2.0 µm and 4.5 µm for acetone and glass, respectively. This behaviour can be explained by the quality of the substrate. P(3HO), being a biocompatible polymer, is an excellent material, which allows for cell proliferation by releasing hydrolysed (*R*)-3-hydroxyacids (Cichoń et al. 2019). These naturally occurring cellular metabolites (compounds also derived from the β-oxidation of fatty acids, so-called ketone bodies) nourish the cell, reducing the need for environmental penetration to search for nutrients. Regardless of the substrate, no differences were noted in the direction of cell movement—fibroblasts moved along random trajectories. The observed characteristics of individual cells’ behaviour on the P(3HO)-based biomaterial correlated well with the movement of the cell monolayer in the in vitro wound healing model (Witko et al. 2020). As previously observed, the cells migrated to the centre of the simulated wound faster on glass than on P(3HO). We compared the rate of healing of artificial wounds with that of another biopolymer—commercially available polylactide (PLA). We designed the experiment with two objectives in mind. First, glass is not suitable for the production of dressing materials, despite having experimentally achieved better parameters for fibroblast monolayer overgrowth, with its curing time equalling 16.2 h. Secondly, PLA is already used in medical applications (Farah et al. 2016). The conducted experiments have shown the superiority of P(3HO) over PLA in stimulating the healing of an artificial wound. The fibroblast monolayer took 16.4 h to close the gap on P(3HO) film, whereas on PLA it was 4.1 h longer.

#### Assessment of biocompatibility of hybrid ceramic-polymer materials

To verify the potential utility of manufactured composite materials in bone tissue regeneration, we conducted in vitro model tests using preosteoblasts from mouse calvaria (MC3T3-E1) (Skibiński et al. 2020). Indirect cytotoxicity tests carried out on extracts after 24-h incubation in the medium, in accordance with ISO 10993, prove the high biocompatibility of the tested materials. The obtained composites did not release toxic substances into the surrounding medium; on the contrary, they favoured the growth of MC3T3-E1, probably through the release of calcium ions alkalizing the environment or the small amount of (*R*)-3-hydroxyacids used by cells as nutrients. In-depth proliferation tests of this cell line on TCP ceramic scaffolds coated and uncoated with P(3HO) were conducted for 7 days. We noticed faster proliferation of preosteoblastic cells on coated than on uncoated materials in the first days of the experiment, suggesting that P(3HO) modulates the implant’s biocompatibility by allowing faster cell adaptation to the substrate. Nevertheless, both materials showed similar effects on cell viability and proliferation to the culture medium-only control.

MC3T3-E1 cells proliferated for several days, and their growth substrates were subjected to microscopic observations by confocal microscopy and SEM. Microphotography of the P(3HO)-coated TCP surface revealed the formation of a uniform cell layer. Using confocal microscopy, we proved that preosteoblastic cells not only cover the implants produced from the outside, but also penetrate the porous structure of the scaffold by anchoring themselves inside its pores. The tests indicated the manufactured bone substitute materials have low cytotoxicity and very high biocompatibility.

### Functionalization of P(3HO) with diclofenac, physicochemical characterization and biological evaluation of the material *in vitro*

Injuries, cuts or invasive surgical procedures often lead to excessive inflammation. To prevent this, anti-inflammatory agents, mainly nonsteroidal anti-inflammatory drugs (NSAIDs), such as diclofenac, are commonly used. Local administration of such substances is necessary to avoid systemic side effects. Unfortunately, simple in situ administration can be dangerous, because exceeding the safe dose can lead to tissue necrosis (Ture et al. 2016). Therefore, administration of the active substance should be done in a controlled manner. Polymeric coatings can successfully serve as slow-release carriers for NSAIDs (Araújo et al. 2017; Maver et al. 2020). Biodegradable polymers such as PLA or PLGA are degraded by volumetric hydrolysis. Meanwhile, PHA polymers mainly undergo surface erosion, which is preferred for many drug delivery applications (Kamaly et al. 2016). Combinations of PHAs with other bioactive substances have already been described (Michalak et al. 2017). To date, short-chain PHAs such as P(3HB) and P(3HB-co-3HV) have often been used for topical delivery of NSAIDs, while PHAs with medium-length side chains have rarely been used for this purpose(Lins at al. 2016). Numerous studies support the anti-inflammatory and analgaesic effects of diclofenac, especially in acute and chronic musculoskeletal pain (Barkin 2015; Wiffen and Xia 2020). This application has already been described in point-of-care delivery systems using polymer-ceramic composites (Zhang et al. 2010; Nikkola et al. 2015; Sidney et al. 2015). However, no studies had reported on the use of P(3HO) as a carrier for diclofenac.

#### Synthesis of diclofenac-functionalized material and its physicochemical characterization

Considering the above-described premises, we aimed to synthesize a P(3HO)-based material that will ensure sustained drug release at the implantation site. Most descriptions have concerned physical mixtures of biologically active substances with polymers (Lins et al. 2016; Michalak et al. 2017); however, such procedures lead to rapid drug release. It is often desirable that the drug is released gradually to provide a stable and long-lasting therapeutic effect. Chemical modification of the polymer by creating stable chemical bonds between the polymer chain and the active ingredient provides a controlled and sustained release. Modifying P(3HB) using p-toluenesulfonic acid (pTSA) as a catalyst to attach a molecule with a carboxyl or hydroxyl bond in its structure has been described (Kwiecień et al. 2015). With pTSA acting on P(3HB) polymer in molten substrates or toluene solution, it is possible to produce cyclic oligomers; further, with a chemical modulator, it is possible to obtain linear oligomers that terminate with ester linkage to the applied chemical compound. Thus, we adapted the developed method for the medium chain modification of P(3HO) (Haraźna et al. 2020). We performed the reactions without a solvent, where the molten substrates—P(3HO) and diclofenac—were reacted at 125 °C for 2 min in the presence of pTSA. After washing off the catalyst, we obtained a viscous yellow liquid, which was subjected to in-depth physicochemical characterization.

^1^H NMR analysis revealed a mixture of three populations of oligomers: linear esters with diclofenac, unmodified cyclic oligomers and linear species terminating with a monomer, in which the hydroxyl group was replaced by a double bond. Diclofenac-modified oligomers dominated the sample—as much as 89% of the diclofenac used was attached. IR spectral analysis showed that modification of the oligomers with diclofenac (Fin-dic-oliPHO) caused IR shifts of the bands responsible for the stretching vibrations of the C–Cl linkages and COO^−^ groups. The modification was also confirmed by X-ray photoelectron spectroscopy. We observed two new signals in the modified material (N 1s and Cl 2p). Comparing the C 1s spectra of P(3HO) and modified oligomers, we observed an increased proportion of carbon atoms involved in ester bond formation (C–O, C = O) which indirectly confirmed the reaction performed.

We used the characterized modifier Fin-dic-oliPHO to prepare polymer blends. Formulations were prepared using half, one and double therapeutic doses of diclofenac (taken as one dose of 0.19 g diclofenac per compound present in the blend) and 0.5Dic-oliPHO, 1Dic-oliPHO and 2Dic-oliPHO were determined, respectively. From thermogravimetric analysis, we observed that the addition of the modifier slightly increased the degradation temperature of the material while maintaining the one-step process. DSC analysis showed an increased glass transition temperature from − 30 to − 29 °C only for 2Dic-oliPHO from the modifier itself, and this was due to its significant amount in the blend (96%). Wetting angle measurements performed for both the modifier and prepared blends with water showed the increasing hydrophilic character of the material with an increasing amount of modifier (viz*.* the wetting angles for P(3HO), 0.5 Dic-oliPHO and 2Dic-oliPHO were 108°, 100° and 83°, respectively).

#### Evaluation of cytotoxicity and biological activity of the produced functionalized materials

**Fig. 4:**
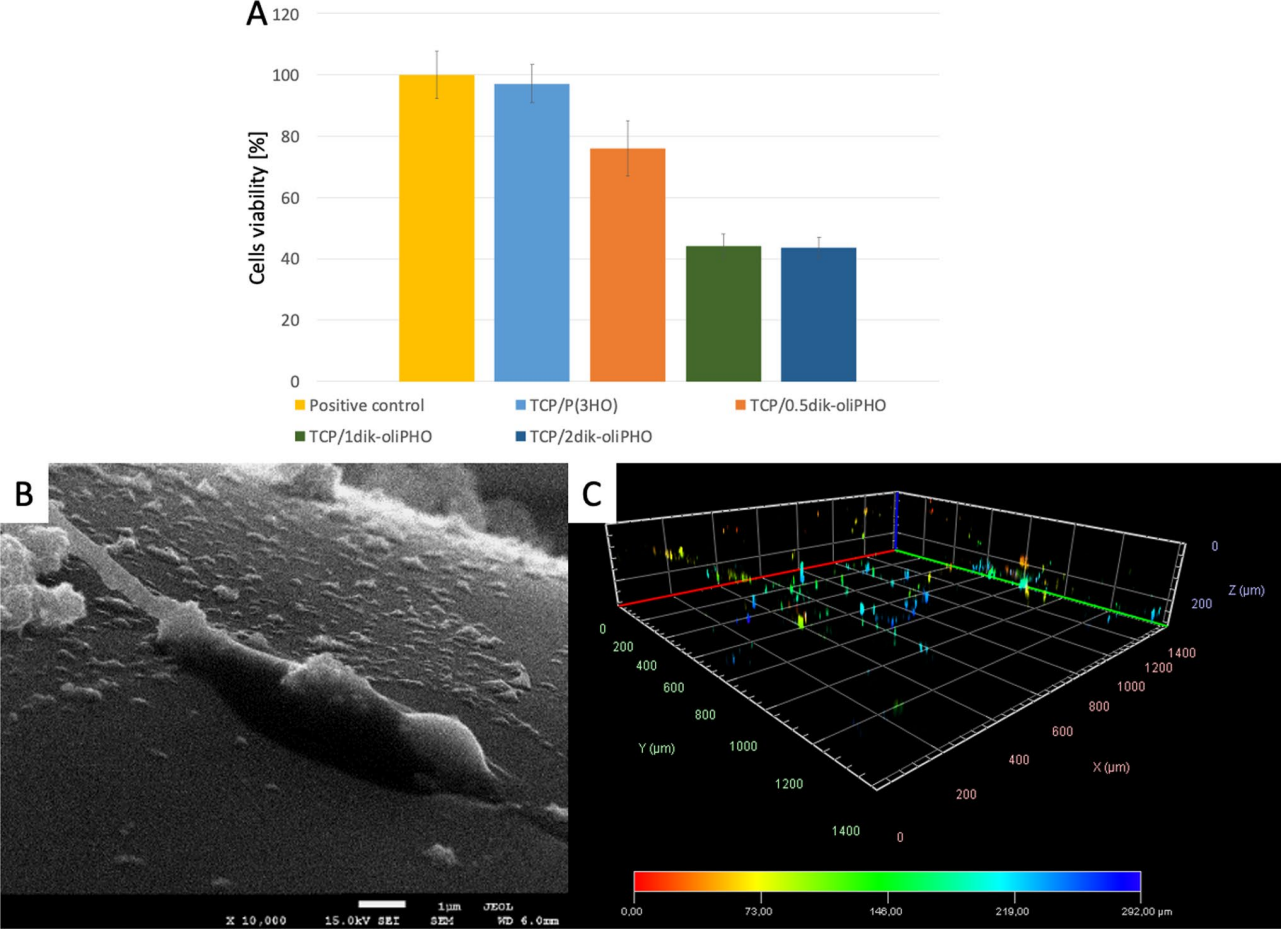
Cytotoxicity of materials towards osteoblasts in the direct contact test on day 7 of the experiment (**A**). Surface of TCP/0.5dik-oliPHO material with visible cells (SEM, **B**). Cell migration into the macropores of the 3D ceramic-polymer matrix scaffold visualized by staining the cell nuclei and observing them using confocal microscopy (**C**) (adapted from Haraźna et al. (2020))

Materials containing an NSAID, such as diclofenac, can be successfully used in atopic or implant applications. We investigated the effect of diclofenac-functionalized P(3HO) on cytotoxicity towards MC3T3-E1 proosteoblastic cells (Haraźna et al. 2020). TCP-based macroporous ceramic scaffolds were coated with blends of P(3HO) together with its oligomers, to which diclofenac at different concentrations (0.5Dic-oliPHO, 1Dic-oliPHO and 2Dic-oliPHO) was chemically attached. Seven days of incubation on the tested composite substrates showed that the material coated with the lowest drug concentration exhibited the lowest cytotoxicity (24 g of modifier per g of blend). Other composites with higher diclofenac content proved to be cytotoxic, significantly reducing proosteoblast viability (Fig. 4A). Through SEM microscopic observations, we confirmed the presence of cells on the surface of the material coated with 0.5Dic-oliPHO (Fig. 4B). Furthermore, confocal microscopy observations allowed us to visualize MC3T3-E1 cells penetrating the interior of the composite (Fig. 4C). Comparing the viability of MC3T3-E1 on initial, unmodified substrates, cells exhibited significantly reduced confluence. They also migrate less into the 3D composites (300 µm versus 500 µm into unmodified material). Together, results showed the materials have promising therapeutic potential. As a continuous exchange of fluids takes place in the human body, the next step before testing these materials in an animal model is the use of dynamic cell cultures with constant flow to simulate the natural movement of physiological fluids, while reducing local accumulation of bioactive substances leached from modified materials.

### PHA depolymerization products and their use for the synthesis of biologically active compounds

Polyhydroxyalkanoates are easily degraded by chemical depolymerization to methyl esters, and further on, by alkaline saponification to a mixture of pure salts of hydroxyacids. The multitude of the (*R*)-3-hydroxyacids building PHAs enables the synthesis of many chirally pure hydroxylated acids containing numerous functional groups, such as ester, nitrile, amine, phenyl and many others (Rhiel et al. 2002; Agnew and Pfleger 2013). It is interesting that microorganisms can synthesize PHA polymers from carbon sources, which structurally do not resemble the chemical structure of the finally obtained monomers (e.g. glycerol, glucose, phenolic compounds are all fermented to biomass, then to P(3HB) or mcl-PHA). These features enable bacteria to synthesize polymers from virtually any carbon source, unlike chemical synthesis that needs appropriate monomers. Moreover, the constant presence of two easily modifiable groups—carboxylic and hydroxylic—makes it possible to use these monomers in broadly understood organic synthesis. (*R*)-3-Hydroxyalkanoic acids provide chiral synthons for the synthesis of pharmaceuticals, vitamins, pheromones and antibiotics (Ohashi and Hasegawa 1992; Choi and Lee 1999; Ruth et al. 2007). For example, Prof. O’Connor’s group at University College Dublin successfully synthesized drugs with increased cancer potential by attaching (*R*)-3-hydroxyacids to a short peptide (O’Connor et al. 2013; Szwej et al. 2015). Another example is the modification of the (*R*)-3-hydroxyl group by exchange with a halogen atom that increases the antifungal and antibacterial activity of tested compounds, showing the potential of PHA monomers as fungal and bacteriostatic compounds (Radivojevic et al. 2016).

One yet unexplored area is the use of PHA monomers to synthesize sugar esters by creating an ester bond between (*R*)-3-hydroxyacid and a selected sugar. Sugar esters are commonly used in food and cosmetic industries due to their surface-active properties and bacteriostatic effect. Another interesting application of (*R*)-3-hydroxyacids is in constructing a new class of organic solvents. PHA monomers with tertiary amines or imidazole derivatives easily form DESs. Sometimes, compounds from renewable sources form a subclass of natural DES (NADES), which show a lack of cytotoxicity to human cells and microorganisms, making them environmentally and human friendly, and quickly biodegradable (Haraźna et al. 2019; Archer et al. 2020). Below, the biocatalytic technique of sugar ester synthesis and DES synthesis based on PHA monomers is described. Next, physicochemical and biological characterization methods will be discussed with the results for both classes of compounds.

### Chemo-biotechnological synthesis of sugar fatty acid esters based on PHA monomers

An interesting potential application of PHA depolymerization-derived monomers is in sugar esters of fatty acids (SFAE) synthesis. These molecules have been known since the 1970s and are commonly used in nutrition, cosmetics and pharmaceuticals (Staroń et al. 2018). First, they allow the synthesis of PHA from carbon sources that do not resemble monomers structurally, which adds value to these substrates (e.g. glycerol, sugars or other biomass components). Second, as previously mentioned, many monomers exist. Third, (*R*)-3-hydroxyacids have a hydroxyl group that enables the design of molecules with new chemical and biological properties, thus creating a comprehensive range of possible syntheses.

We developed a workshop for the effective synthesis and analysis of SFAE (Snoch et al. 2019b). We used lipases, which can enantioselectively esterify selected acyls to sugar molecules. At the screening stage, methyl nonanoate and glucose were chosen as model substances, greatly simplifying the experimental setup and allowing for comprehensive testing of reaction conditions easily comparable to the results described in literature. The first SFAE synthesis tests showed that the immobilized enzyme allows a greater percentage conversion than free enzymatic formulations. Thus, we decided to construct our own immobilized preparations based on hollow silica microspheres, which can be successfully used in flow reactors without causing a significant increase in pressure. We used two types of silica substrates: with –OH and –NH_2_ functionalizations. Enzymes were connected using divinyl sulfone and glutaraldehyde linkers, respectively. The prepared spheres were reacted with EL070 lipase, and as controls, we used commercially available and immobilized lipases from *Candida atharctica* (CalB) and *Thermomyces lanuginosus* (TL-IM). To dissolve both reagents (namely methyl nonanoate and glucose), we tested many solvents, of which 2-methyl-2-butanol with DMSO (8:2) was the most suitable. In the flow reactor, the most effective way to synthesize SFAE was catalyzed by spheres with –NH_2_ functionalization, with 88.3% conversion in the second passthrough. Parallel to the work on SFAE synthesis, we developed a rapid analysis method using the HPLC–MS/MS system. To separate products from substrates, reverse-phase liquid chromatography in a water–methanol gradient was used. Detection of fragmentation ions of the analysed compounds, preceded by ESI-type ionization, allowed precise qualitative and quantitative analysis of the compounds.

We conducted further research aiming at structural modification of PHA monomers before the final SFAE synthesis (Snoch et al. 2019a). As a goal, we chose hydroxyl group modification, enabling the introduction of a fluorine-containing group into the molecular structure. Halides, including fluorine, confer different chemical and biological properties to the initial compounds without these atoms. With colleagues from the Maj Institute of Pharmacology Polish Academy of Sciences, we conducted screening tests using methyl esters from two types of PHA (polyhydroxynonanoate, P[3HN]; polyhydroxyphenylvalerate, P[3HPV]). We tested many reaction media, nucleophiles and modifiers, including 1-fluoro-3-iodopropane, 1,1-difluoro-2-iodoethane and 1,1,1-trifluoro-3-iodopropane. We successfully synthesized new P(3HN) and P(3HPV) monomer derivatives containing the trifluoroethoxylated group instead of the ‘original’ hydroxylated group. Using the developed biocatalysis workshop, we created a SFAE library based on glucose and unmodified and modified P(3HN) and P(3HPV) monomers. With cooperation from scientists at the Medical University of Warsaw, these compounds were tested for their anti-microbial potential. The assays on the panel of 15 strains (Gram-positive and negative bacteria, and yeasts) allowed us to determine the minimum inhibitory concentration for each synthesized SFAE to these organisms. Glucose esters and PHA monomers in the mentioned studies showed moderate anti-microbial activity.

### Synthesis and characteristics of a subclass of ionic liquids—deep eutectic solvents—based on PHA monomers

Nowadays, alternatives to conventional solvents based on petroleum processing products are increasingly being sought. DESs are increasingly becoming used as alternatives. These mixtures can be successfully used in electrochemical processes, polymer synthesis, biocatalytic processes or extraction of biomass components (Haraźna et al. 2019). DESs are made of a hydrogen bond donor and acceptor, and their melting point is usually below 100 °C. They may contain molecules such as organic acids, sugars, chlorides of quaternary amines and imidazole compounds.

PHA monomers have two functional groups, hydroxylic and carboxylic, which can serve as donors for hydrogen bond formation. Considering these features, we conducted the first synthesis of DES based on PHA monomers (Haraźna et al. 2019; Archer et al. 2020). We used derivatives of two polymers—P(3HN) and mcl-PHA—synthesized from rapeseed oil fatty acids. The former has two monomers—(*R*)-3-hydroxynonanoic and (*R*)-3-hydroxyheptanoic acids—at a ratio of 7 mol:3 mol. This mixture has been used for ‘proof-of-concept’ experiments testing the possibilities of synthesizing DESs from 3-hydroxyacids (Haraźna et al. 2019). We showed that P(3HN) monomers successfully form liquids with choline chloride ([Ch]Cl), 1-ethyl-3-methylimidazole chloride ([EMIm]Cl) or tributylmethylammonium chloride ([TBMA]Cl) hydrogen bond donor:recipient ratios of 1:1 to 2:1. The control mixture created from aliphatic nonanoic and hexanoic acids to mimic a mixture of P(3HN) at a ratio of 1:1 with choline chloride did not create a liquid at room temperature compared to an identical mixture from P(3HN). Thus, we hypothesized that the presence of additional hydroxyl groups enables the synthesis of DES-type liquids when the aliphatic chain of carboxylic acid is longer than 9 carbons. To confirm this hypothesis, we used a mixture of monomers originating from mcl-PHA obtained from rapeseed oil fatty acids to construct DES based on choline chloride. This mixture contained the following (*R*)-3-hydroxyacids: C6, C8, C10, C12, C12:1 and C14 at respective molar ratios of 4.0:33.7:31.8:5.0:6.4:19.1. We successfully obtained a series of DES based on choline chloride with a mixture of mcl-PHA acids (respective ratios of 1:1, 1.5:1, 2:1, 2.5:1 and 3:1), thus confirming the hypothesis (Archer et al. 2020).

Using nuclear magnetic resonance and Fourier-transform infrared spectroscopy, we confirmed that PHA monomer DESs with tested acceptors were purely physical mixtures. These techniques also allowed us to demonstrate the formation of hydrogen bonds between mixture components by observing the appropriate signal shifts. We observed that the more PHA monomers in the mixture, the greater the spectral shifts, which means the creation of stronger intermolecular interactions (Haraźna et al. 2019). Subsequent research allowed deeper knowledge regarding the mechanisms of hydrogen bond formation in PHA monomer–based DESs (Archer et al. 2020). Using quantum–mechanical calculations in the gas state, we studied choline-chloride-(*R*)-3-hydroxyhexanoic acid complexes, where the share of acid was gradually increased. We determined the initial strength of the hydrogen bonds. First, the hydroxylic and carboxylic monomer groups formed an intramolecular hydrogen bond (–OH component interaction), where = O in the carboxylic group forms a hydrogen bond with the –OH group of choline either directly or through the chlorine present in the complex. This atom constitutes a central ion, which is a specific ‘bridge’ between acid and choline in the production of hydrogen bonds in complexes with an increased number of monomers.

Regardless of the hydrogen bond acceptor used, the synthesized liquids exhibited a density close to unity (g/cm^3^ at 60 °C) without any visible correlation. All DESs based on PHA monomers are highly viscous liquids. The amount of monomer used in their construction reduces the mixture viscosity non-linearly, and there is no linear dependence of the liquid viscosity on temperature (non-Arrhenius dependence). The obtained DSC results collected for synthesized DESs in the temperature range of − 60–60 °C had almost-flat lines, suggesting no vitreous transitions in the tested temperature range. In contrast, DES mixtures had a complex thermal degradation mechanism. Preliminary tests only allowed a rough determination of this process. Based on the courses of TGA curves, we speculate that degradation begins with the evaporation and degradation of organic acid components, before decomposition of the hydrogen bond acceptor. Further, we showed that synthesized eutectic mixtures, to some extent, mix with slightly polar solvents (methanol, chloroform), but not with highly polar (water) and non-polar (n-hexane) solvents. Correlating these results with the measurements of wetting angles obtained for DESs based on choline chloride and mcl-PHA monomers (mica ~ 22°, Teflon ~ 48°), we conclude that these liquids are polar and slightly hydrophobic.

An interesting application for synthesized liquids may be in biomass fractionation. Solubility tests of individual wood biomass components showed that PHA monomer–based DESs selectively dissolve lignin and not cellulose, indicating their potential use in biorefinery. Moreover, biodegradation tests of all DESs suggest that those based on choline chloride and PHA monomers are environmentally friendly. In the tested active sludge system, they were fully biodegradable within 6 days. An additional advantage of these solvents is the lack of cytotoxicity. The viability of fibroblasts falls below 80% only above the concentration of 500 mg/L DES, based on choline chloride and P(3HN) monomers.

All preliminary studies on the synthesis and characterization of new PHA monomer–based deep eutectic mixtures and other natural components, such as choline chloride, open the way to new perspectives on their application. The variety of bacterial monomers with natural H-bond acceptors creates many possibilities for the synthesis of new, tailor-made biodegradable and nontoxic DES compounds with specific physicochemical properties for the desired application.

## Conclusions

Polyhydroxyalkanoates are very attractive materials, not only because of their diversity, biocompatibility and biodegradability, but also because their monomers are a source of bioactive synthons. The potential of PHA as a material for medical applications opens the way for the synthesis of useful new solutions for the construction of dressings, implants and other biomedical materials. The presented synthesis methods and characteristics of functionalized PHA polymers—or small, biologically active molecules based on PHA monomers—constitute a point of reference for solutions to meet the expectations of this huge market. The developed methods of synthesis of either polymers or new solvents based on them, combined with the polymers/chemicals themselves, are in line with the objectives set for the United Nations Sustainable Development Goals (SDGs). For example, these include SDG 3 Good health and well-being; SDG 9 Industry, innovation and infrastructure; SDG 11 Sustainable cities and communities; SDG 12 Responsible consumption and production; SDG 13 Climate action; and finally SDGs 14 and 15 Life below water and on land. The conducted research clearly indicates the positive environmental and economic impacts promised by PHA, showing the possibilities of synthesizing these polymers and their derivatives in future biorefinery solutions.
